# What Is a Group? Young Children’s Perceptions of Different Types of Groups and Group Entitativity

**DOI:** 10.1371/journal.pone.0152001

**Published:** 2016-03-24

**Authors:** Maria Plötner, Harriet Over, Malinda Carpenter, Michael Tomasello

**Affiliations:** 1 Max Planck Institute for Evolutionary Anthropology, Leipzig, Germany; 2 University of York, York, United Kingdom; 3 University of St Andrews, St Andrews, United Kingdom; University of Warwick, UNITED KINGDOM

## Abstract

To date, developmental research on groups has focused mainly on in-group biases and intergroup relations. However, little is known about children’s general understanding of social groups and their perceptions of different forms of group. In this study, 5- to 6-year-old children were asked to evaluate prototypes of four key types of groups: an intimacy group (friends), a task group (people who are collaborating), a social category (people who look alike), and a loose association (people who coincidently meet at a tram stop). In line with previous work with adults, the vast majority of children perceived the intimacy group, task group, and social category, but not the loose association, to possess entitativity, that is, to be a ‘real group.’ In addition, children evaluated group member properties, social relations, and social obligations differently in each type of group, demonstrating that young children are able to distinguish between different types of in-group relations. The origins of the general group typology used by adults thus appear early in development. These findings contribute to our knowledge about children's intuitive understanding of groups and group members' behavior.

Young children grow up in a complex social world in which they are constantly flooded with social information. Our social world is composed not only of individuals but of an array of different relationships and social groupings. One challenge for children is to decipher which of these social groupings are meaningful. People can appear to be a group from the outside, for example simply because they are in close proximity to each other, but they can be connected with each other at different levels: they can be kin or friends, be on the same sports or work team, be part of the same national or language group, or they can be associated with each other only briefly and loosely when, for instance, they take the same bus to get to the airport, or line up at a counter at the same time. Determining the type of group to which an association of people belongs is not only crucial for being able to understand individual group members’ behavior but can also be a short-cut to predicting how group members will relate to each other. For example, one can expect kin or friends to be loyal to each other, but one might not expect this about people who happen to be lining up at a counter at the same time. Another important form of predictions that can be drawn from social groupings, but which has been understudied in previous research (see also [1]), regards the grouping as a whole. For example, a friendship is supposed to be a longer-lasting, more coherent entity than a gathering in front of a counter.

When it comes to the perception of social groupings, Lickel and colleagues [2] have argued that adults apply a folk typology, in which they intuitively distinguish between four qualitatively different types of groups. In support of this idea, Lickel at el. [3] investigated how adult participants sorted 40 examples of real-life groups, and how they rated each of these groups on a set of eight group characteristics such as shared goals, similarity of group members, interaction among group members, and group size. They found that participants distinguished four basic types of groups: *intimacy groups* (such as families and friends), *task groups* (such as work or sports teams), *social categories* (such as women or U.S. citizens), and *loose associations* (such as people waiting in line at a counter). Participants associated different group characteristics with each group type, for example a long duration and high levels of interaction for *intimacy groups*, common goals and interaction in *task groups*, large size and member similarity for *social categories*, and short duration and low levels of similarity and common goals for *loose associations* (for an overview, see [2]). Related research has shown that adults treat some social groupings as entities [4–6]. The extent to which a group appears to be a coherent entity and therefore possesses a quality of “groupness” has been referred to as “entitativity” [2–5, 7]. Lickel and colleagues showed that the four types of groups were perceived by adults to have different levels of entitativity, with the highest level for *intimacy groups*, followed by *task groups*, *social categories*, and *loose associations*.

This group typology has received further support and validation from work in anthropology [8, 9]. Interdisciplinary work has linked these different types of groups to different relational models that are more or less prominent within each group type [10]. For example, communal sharing, a relationship in which I see “what is mine as yours” is more pronounced in *intimacy* groups than in other types of groups. It has been argued that children do not develop a fully-fledged concept of these different relational models before nine or ten years of age [8, 9].

Despite the theoretical importance of this group typology, very little research has investigated its origins in childhood. Instead, developmental research on group cognition in young children has focused mainly on children’s in-group biases, that is, their preference for members of their own group over members of other groups. Research in this tradition has shown that children prefer members of their own group on a variety of implicit and explicit measures [11–14]. Another line of research focuses on the inferences children draw about individuals based on their group membership. For example, 4- to 6-year-old children predict what a person will do, like, or intend on the basis of that person’s gender, race, or ethnicity [15–17]. Children also use information about group membership to make inferences about social interactions: Knowing that two individuals are either from the same or from two different groups influences their prediction about whether those individuals will harm each other (around 4 years; [18]), help each other (from 6 years; [18]), or be friends with each other (from 7 years; [19]).

However, this body of research leaves at least three significant gaps in our knowledge about children’s understanding of groups. First, previous research has focused primarily on just one type of group: the one Lickel and colleagues refer to as *social categories*, thus limiting what we can conclude about children’s understanding of group relations more generally (although see, e.g., [7, 20, 21], for work on preferential behavior towards intimacy and task group members). Second, the main focus of this previous research has been on children’s attitudes and expectations about in-group as compared with out-group members. However, as illustrated in our introductory examples, relationships among members of an in-group may differ in systematic ways depending on the type of in-group to which they belong. Finally, previous work has focused mainly on children’s perceptions of and expectations about individual group members rather than on their perceptions of and expectations about the group as a whole. It is thus important for our understanding of the development of group psychology to ask whether children distinguish different types of social groups and whether they expect relationships within and characteristics of these types of groups to differ from each other.

One exception to this general trend is a study conducted by Svirydzenka and colleagues [7]. They found that 10-year-old children intuitively distinguish the same four main types of groups as adults: *intimacy groups*, *task groups*, *social categories*, and *loose associations*. They also judged the level of entitativity of different group types in similar ways as adults, but their assessments seemed to rely on group characteristics that were more perceptually salient (for example the level of interaction) than adults, who focused on more abstract features such as the importance of the group for its members [22].

Inspired by this study and Lickel and colleagues’ work [3], we investigated whether the origins of this folk theory of groups could be seen even in children as young as 5 to 6 years of age. This is an important age in the development of group cognition as 5 to 6 years appears to be just at the border of explicit group understanding. It is at this age that children first show a more general preference for in-group members, even in more abstract and novel groups (in the minimal group paradigm; [21, 23]). Furthermore, it is also at this age that children first become able to predict intergroup relations in third party contexts at least for social categories (e.g., [16, 18]).

Thus our objective was to investigate whether, in addition to these preferences and expectations, children of this age also have a more general understanding of groups and different types of group–in other words, an early folk typology of groups. Several prominent theoretical accounts of the origins of intergroup psychology postulate substantial development between the age group in our study and the youngest age, so far, at which a group typology has been found, 10 years [24–26]. However, given their relatively sophisticated abilities in other areas of group cognition, we predicted that already by 5 to 6 years of age, children would be able to make subtle distinctions between different types of groups and use this understanding in order to make inferences about group members’ behaviors within different group types.

As a first step, we measured children’s spontaneous definition of a group. We did this to investigate children’s naïve, spontaneous ideas about groups, before presenting them with different group types. We predicted that children would be able to give some appropriate examples of groups and were especially interested in whether they would focus on one particular example or definition when thinking about groups (e.g., mention just one group type), or whether they would be able to give a more abstract definition (covering all group types, such as “a collection of people”). Second, because recent work has shown that 5-year-old children have comparable preferences for two types of group members–task group members and social category members [21]–we investigated which of these two examples (operationalized as people who work together vs. people who are similar to each other) children thought was most representative of a group. Third, we investigated whether preschool children would see an *intimacy group*, a *task group*, a *social category*, and a *loose association* as qualitatively different.

It was impossible, given the young age of our participants, to adopt the exact methods of previous studies, which used complex tasks such as sorting of examples of groups and rating multiple group characteristics for each example. To simplify the procedure so that young children would understand it, we thus created a prototype for each of the four types of groups and asked children to judge these prototypes on entitativity and 12 other group characteristics. These group characteristics were generally inspired by the characteristics Lickel et al. [3] and Svirydzenka et al. [7] chose. However, in addition, we asked about several further characteristics that are important topics in recent work on the developmental origins of group psychology (e.g., [20, 27–29]) and anthropology [8, 9]. There were four main sets of group characteristics. The first three involved judgments and predictions about individual group members and group member relationships (see, e.g., [27]). The first set involved judgments about social obligations and prosocial behaviors among group members (helping, sharing, and loyalty; e.g., [18, 20, 28, 30]). The second involved the quality of group members’ social relationships (liking, familiarity, interdependence, and joint goals; [7, 31]). The third involved properties marking fundamental similarities among group members (group member similarity, shared preferences, and common knowledge; [29, 32, 33]). The fourth set, in contrast, involved traits of the group itself, concerning characteristics that apply to the group as a whole, rather than to individual members (permeability, continuance, and entitativity; [3]). We predicted generally that children’s perceptions of and expectations about groups would be contingent upon the type of group they were presented with and that they would recognize that a loose association was not a real group.

## Method

### Ethics statement

The present study strictly adhered to the legal requirements of the country in which it was conducted, and a detailed procedure was approved in advance by the Ethics Committee of the department in which it was conducted. In addition, parents of all children who participated in the study gave informed written consent.

### Participants

Participants were 48 5- to 6-year-olds (*mean =* 6 years, 0 months, 5 days; *range* = 5 years, 0 months, 3 days to 6 years, 10 months, 8 days) from a medium-sized city in Germany. Half of the participants were female. Children were tested in their kindergarten. One additional boy was tested but excluded from analyses due to extended interruption of his test session because of the distraction caused by the noise level outside the testing room.

### Study Materials

**Fig 1:**
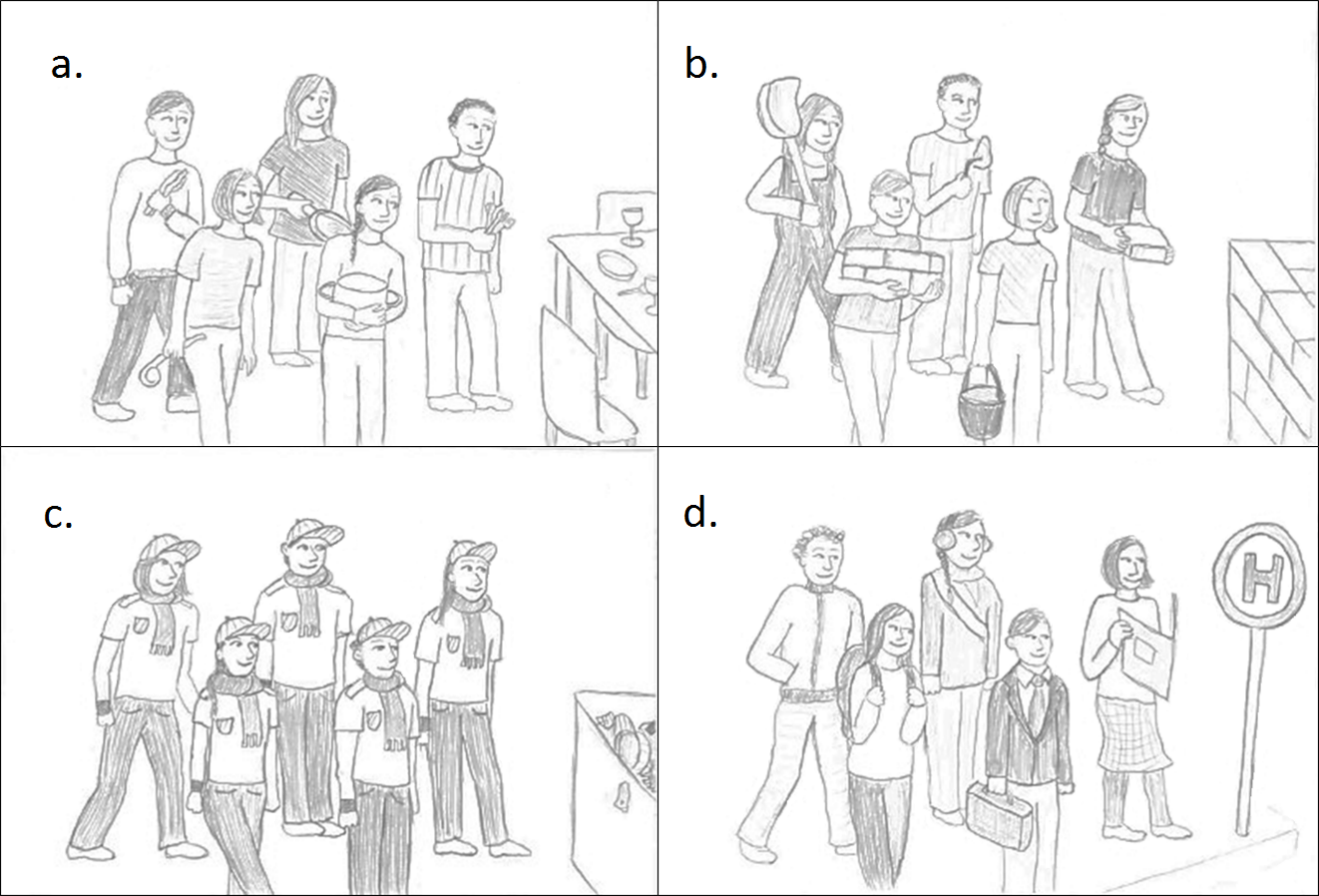
Study materials. Pictures for prototypes of (a) an *intimacy group*, (b) a *task group*, (c) a *social category*, and (d) a *loose association*.

Children were presented with drawings (12.5 x 9.5 cm each) of four groups attached to a 30 x 21 cm piece of cardboard. Pictures were arranged in two rows; their positions were counterbalanced, using 12 different arrangements (see Fig 1 for one version). Friends were chosen as the prototype for *intimacy groups*, people who are building a house for *task groups*, people who look alike for *social categories*, and people who are waiting at a tram stop for *loose associations*. Each picture showed five individuals, three females and two males, casually arranged in two rows and facing toward the front right. The position of males and females and their hair styles (straight vs. curly for the males; short, long, or ponytail for the females) were counterbalanced across pictures.

An initial pilot phase with 17 additional children confirmed that 5- to 6-year-olds understood the verbal questions and the pictorial stimuli.

### Design and Procedure

Children were tested in a quiet room in their kindergarten. After a brief conversation, which served as a warm-up phase, the child and the experimenter sat at a table.

Before presenting any pictures, participants were asked about their spontaneous definition of a group. The experimenter asked two open questions: (1) “What is a group?” and, since piloting had revealed that most children understood the word “group” only as kindergarten group (i.e., class), the experimenter always asked (2) “And besides kindergarten groups, do you know any other groups?”

Following this, still without any pictures present, children were asked, “What is a better example of a group: people who work together or people who are similar to each other?” The order of the two examples was counterbalanced.

The four pictures were then brought out and introduced by the experimenter as follows (in the order in which they were displayed on the piece of cardboard):

1. Intimacy group (friends): “These people here are friends. Look, they’re all just about to eat lunch.”
2. Task group (people building a house): “These people here are building a house. Look, they’re all just about to go on working on it.”
3. Social category (people who look alike): “These people here look alike. Look, they’re all wearing the same outfits.”
4. People at the tram stop: “These people here are each waiting for a different tram. Look, they all happen to be waiting at the same tram stop.”

Children then were asked to point at the pictures which showed a real group (“Which ones are real groups?”; group entitativity, trial 1).

The experimenter then asked questions about 12 group characteristics, asking children to point out the group that was most likely to have a particular feature. Children were asked about helping (“In which picture do people help each other most?”), sharing (“In which picture do people share their things with each other?”), loyalty (“In which picture should people not leave each other?”), liking (“In which picture do people like each other most?”), familiarity (“In which picture do people know each other best?”), interdependence (“In which picture do people need each other the most?”), joint goals (“In which picture do people want to do something all together?”), similarity (“In which picture are people most similar to each other?”), shared preferences (“In which picture do people like the same things?”), common knowledge (“In which picture do people know the same things?”), the groups’ low permeability (“In which picture can’t one join easily?”), and lack of continuance (“In which picture will the people not meet again?”). After each question, children were asked why they chose that group. Children were asked the 12 questions in counterbalanced order, using a 12 x 12 Latin square design; that is, there were 12 different order sets, with each question in each position exactly once. The 12 picture arrangements were randomly assigned to the 12 question order sets. Each combination was tested both with a male and a female participant.

At the very end, children were again asked to point at the pictures which show a real group (group entitativity, trial 2) to investigate whether the evaluation of the 12 group characteristics would influence participants’ entitativity ratings.

### Coding and Reliability

Children’s responses were coded from videotape. Children’s combined answers to the first two questions about their definition of a group (i.e., “What is a group?” and “Besides kindergarten groups, do you know any other groups?”) were coded in one of three hierarchical categories from most abstract to most specific. The most abstract category was coded when children gave a general, overarching definition of a group as a social collective, that is, if they defined a group as a collection of people, (e.g., “People who belong together”). A middle category between the most abstract and specific definitions was coded when children defined a group as a collection of specific individuals, that is, as a collection of children, (e.g., “Many children”). Participants never gave definitions of a group as a collection of specific individuals other than children. The most specific category was coded if children gave one concrete example of a group (e.g., a kindergarten class label). If children gave no answer or answers that fell in none of these categories (e.g., “Where one can play”) they were coded as the fourth category “other.” If children gave more than one answer, they were given credit for their most abstract definition.

For the questions “Which ones are real groups?” (group entitativity, trial 1+2), it was coded first which picture(s) were chosen. For a follow-up analysis, scores were then given for the order in which children chose the pictures they thought were groups. For each child, the picture that was chosen first was scored with the value 4, the second choice was scored with 3, the third with 2, and the fourth with 1. If a picture was not chosen by a child, it was scored zero.

For the 12 group characteristics questions, it was coded which picture children chose. If children did not choose any picture, or said “I don’t know,” this choice was coded as blank (resulting in some N’s < 48).

Twenty-five percent of the data (12 children) were randomly chosen to be independently coded by a second rater who was unaware of the aims of the study. Agreement between the two coders was excellent (all Cohen’s κ’s > .994).

## Results

For all analyses, an equal split of the sample into a subset of 5- and a subset of 6-year-olds, as well as into boys and girls, revealed a similar pattern of results, with no significant differences between the age and gender groups except for a significant gender difference for the group characteristic question on interdependence. There, girls (*n* = 8/24) were more likely than boys (*n* = 2/24) to say that friends are interdependent. However, they did not do so significantly above 25% chance level, thus we consider this a minor difference. We thus collapsed across the factors gender and age for the analyses reported below.

### Definition of a “Group”

First, children’s combined answers to the open questions (1) “What is a group?” and (2) “Besides kindergarten groups, do you know any other groups?” were investigated (see Table 1). The main finding was that very few children (only 8.3%) gave an answer indicative of a more abstract definition of a group as a collection of people. If a collection of children is added to this, the number rises to 52.1%. Thirty-seven percent of participants gave very specific, concrete examples of groups to define what a group was, and all of the examples children gave were kindergarten groups. Despite the fact that we specifically asked them to give examples of groups besides kindergarten groups, no child, including those whose first description met the most abstract category, could give a concrete example of a group besides kindergarten groups. Thus, although almost half of the participants could give a more or less abstract definition of a group, all examples they could think of spontaneously were limited to kindergarten groups.

**Table 1:** Percentage of children who gave each type of answer to the questions “What is a group?” and “Can you think of any other group besides kindergarten groups?” coded in hierarchical categories from most abstract to most specific.

% (N = 48)	Coding categories from most abstract to most specific	Examples of children’s responses
8.3%	A collection of people	“Many people,” “People who belong together,” “Made up of people”
43.8%	A collection of children	“A lot of children,” “Children who are together,” “Many babies, or preschoolers”
37.5%	Concrete example(s) of groups	All examples were kindergarten groups, i.e., group labels from their kindergarten (e.g., “The butterflies,” “The flowers”) or “In a kindergarten”
10.4%	Other/ No answer	“Where one can play,” “A room,” “Where one has to get dressed”

Next, we analyzed which of the two examples given children chose as the better example of a group. Most children (80.9%) chose “people who work together” as the better example of a group; the remaining 19.2% chose “people who are similar to each other.” This difference was significant (binomial test, *p* < .01; all reported *p* values are two-tailed).

### Group characteristics

#### Group entitativity

In the first entitativity trial we investigated which of the four pictures children perceived as depicting a “real group.” As predicted, most of the children perceived friends (85.4%), people building a house (81.3%), and people who look alike (85.4%) as real groups. In contrast, only 33.3% of children perceived people at the tram stop as a real group. This difference was significant, χ^2^(3, *N* = 137) = 13.04, *p* < .01. The second trial at the end of the session revealed almost identical results (friends: 89.6%, people building a house: 85.4%, people who look alike: 89.6%, people at the tram stop: 35.4%; χ^2^(3, *N* = 144) = 13.44, *p* < .01). Thus young children are able to accurately distinguish groups from mere collections of people.

In order to investigate whether children perceived any of the examples as more typical of a group than others, in a follow-up analysis we investigated the order in which children chose the pictures in the first trial. We reasoned that, if children view one type of group as a particularly good example of a ‘real’ group, then they should choose it first. A Friedman test revealed a significant difference between the four pictures’ scores (χ^2^ = 31.54, *df* = 3, *p* < 0.01). A post-hoc analysis using the R-package “pgirmess” [34] revealed that this effect was driven by a lower order score of the picture “people at the tram stop” (*M* score = 0.88, *SD* = 1.38) compared to each of the other three pictures. That is, “people at the tram stop” was least often chosen to be a real group or else was chosen later in the sequence. There were no pairwise differences between the pictures “friends” (*M* = 2.58, *SD* = 1.35), “people building a house” (*M* = 2.10, *SD* = 1.31), or “people who look alike” (*M* = 2.85, *SD* = 1.44), showing that children did not choose any of these pictures more often, or earlier in the sequence, than the others. This suggests that children see these three categories as equally representative of a real group. Again, the same pattern of results was replicated for the second trial (χ^2^ = 36.78, *df* = 3, *p* < 0.01; with “people at the tram stop” differing from the other three pictures in pairwise post hoc analyses).

#### Group characteristics questions

Finally, children’s answers to the questions about the 12 remaining group characteristics were analyzed. Since the justifications children gave for their answers were often circular (e.g., “Friends share with each other because they are friends,” or “People who look alike like the same things because they look alike”) or otherwise unhelpful, we focused on children’s choices. To avoid problems associated with multiple testing, we performed a downward analysis of the data before approaching the actual research question statistically [35]. As a first step, to see if the pattern of children’s choices differed significantly from random choices, a permutation test was computed [36, 37]. For that, random choices were simulated by permuting the original choices within each participant over all questions 1000 times. Permuting the choices within each participant controls for a participant’s potential preferences for a particular picture and controls for non-independence of data (i.e., that participants provided multiple choices across all questions). After this, chi-square tests across all responses were conducted for all these permutations as well as the set of original data. To get an estimate of a *p* value as an indicator of whether the original choices were significantly different from chance, the proportion of permutations that revealed a chi-square test statistic at least as large as that of the original data (χ^2^ = 304.36) was estimated, revealing *p* = .001. The distribution of children’s choices in the original data thus differed significantly from a random distribution.

After having established that participants’ responses were different from a random distribution, post-hoc analyses were conducted to investigate what was driving the differences [35]. The first post-hoc analysis focuses on each individual question (i.e., the rows of Table 2) by calculating chi-squares for each question, to see which of the 12 questions revealed a response pattern differing from chance. It turned out that all group characteristics questions did so (all *p*’s < 0.03). Thus children showed significant preferences for which pictures to choose in response to each particular question. This finding again allowed us to follow up and investigate which picture was chosen most often for each group characteristics question. Binomial tests for each individual choice were conducted (i.e., the cells of each row in Table 1; [35]), testing against chance level (0.25). Choices that were made significantly above chance are in bold in Table 2 (all *p*’s < 0.01). As predicted, there were systematic differences in how children expected members of the different types of groups to relate and interact. They expected friends to like each other, share with each other, and be loyal to each other. They expected people who build a house together to be interdependent, and to help each other. They expected people who look alike to be similar, familiar with each other, and to share common knowledge and similar preferences. In contrast, children characterized a collection of people who stand at the tram stop as low in permeability (that is, difficult to join), and as not continuing in the future.

**Table 2:** The percentage of children who chose each picture for each group characteristics question.

	Intimacy group (Friends)	Task group (People building a house)	Social category (People who look alike)	Loose association (People at the tram stop)	*n*
Obligations and prosocial behaviors					
Helping	20.8%	**68.8%**	6.3%	4.2%	48
Sharing	**64.6%**	14.6%	14.6%	6.3%	48
Loyalty	**43.5%**	26.1%	23.9%	6.5%	46
Nature of relationships					
Liking	**43.8%**	27.1%	16.7%	12.5%	48
Familiarity	36.2%	17.0%	**42.6%**	4.3%	47
Interdependence	20.8%	**54.2%**	18.7%	6.3%	48
Joint goals	31.4%	35.4%	27.1%	6.3%	48
Similarities between group members					
Similarity	4.3%	10.6%	**59.6%**	25.5%	47
Shared preferences	11.1%	33.3%	**51.1%**	4.4%	45
Common knowledge	22.7%	9.1%	**56.8%**	11.4%	44
Characteristics of the group as a whole					
No continuance	12.8%	6.4%	17.0%	**63.8%**	47
Low permeability	10.9%	26.1%	15.2%	**47.8%**	46

Since all children who made a choice (indicated by the n) chose just one picture for each question, rows add up to 100%. Choices that were made significantly above chance (25%) are in bold (binomial tests, all p’s < 0.01).

## Discussion

This study investigated children’s general understanding of groups and their perceptions of different types of groups, a topic that so far has been understudied in developmental research. We investigated the naïve conceptions young children have about groups and examined whether children show distinct patterns of judgments and expectations regarding groups’ and group members’ characteristics across four different key types of groups.

There were several interesting findings in this study. First, we found that when asked “What is a group,” only a small minority of children (8.3%) were able to define a group abstractly and generally as a collection of people. The vast majority of children defined a group as a collection of children or by giving an example of a kindergarten group. None of them could think of any concrete examples for a group beyond kindergarten groups. Thus, children do have some understanding of what a group is; however their understanding is limited in that other types of groups do not spontaneously come to mind for children as readily as they might for adults.

Second, when asked to choose which is the better of two given examples of a group, a large majority of children chose people who work together over people who look similar. That is, although children generally assume group members to be similar to each other in third-party contexts [29, 32], when forced to choose between the two types of groups, groups based on collaboration may be seen as stronger examples of groups than groups based on similarity for young children. This is an interesting finding because previous accounts have usually stressed perceptual salience, such as group markers, in children’s concepts of groups (e.g., [38]). However, a recent theoretical account from evolutionary anthropology suggests that social connections based on collaborative activities are more deeply rooted than those based on group markers indicating similarity [39]. Thus it would be useful for future studies to further investigate children’s understanding of and expectations about social groups that have collaborative roots.

Third, children’s judgments and expectations about four different types of groups and their group members were examined. We found that a large majority of children judged an *intimacy group*, a *task group*, and a *social category* to be real groups. The entitativity judgments for each of these groups were almost identical, that is, children thought that each of these three types of groups forms a coherent unit to the same degree. Only the *loose association* was judged as being significantly lower in entitativity, and thus as qualifying less as a real group. Adults and 10-year-olds in previous studies [2, 7] judged the entitativity of *loose associations* to be lowest as well, but in contrast differentiated the entitativity levels of the first three group types: They rated entitativity highest for *intimacy groups*, followed by *task groups*, and *social categories*. This finding thus reveals an interesting developmental pattern suggesting that, compared to adults and older children, young children show a less fine-grained perception of group entitativity.

However, a fourth set of findings showed that children did have a relatively sophisticated understanding of the unique pattern of group characteristics associated with each group type. This is an important contribution to the literature, as it shows that children distinguish different types of in-group relations from each other. Children perceived the *intimacy group*, *task group*, and *social category* as well as the *loose association* to have different patterns of group traits and they judged that group members of these different types of groups would have different kinds of characteristics, relationships, and obligations to one another. For example, children judged the *intimacy* and *task group* members to have social obligations and to behave prosocially towards one another. In particular, friends were judged to like, share with, and be loyal to each other, and people building a house together were perceived to be interdependent and help each other. Children’s judgments thus correspond well with adult intuitions about the members of these two types of groups, in that *intimacy groups* typically involve positive, long-lasting, reciprocal relationships [40] with a focus on communal sharing [10], and *task groups* possess basic qualities of cooperative interactions: interdependence and mutual help [39, 41]. In addition these findings suggest that children's judgments about different types of groups correspond well to the way they behave toward members of these groups themselves. For example, preschoolers share and direct others to share more with *intimacy group* members [20, 42], and they readily and preferentially help their *task group* members [21, 43] and are sensitive to their interdependence with them [44, 45]. Children judged the *social category* members to be familiar with each other and to possess properties marking fundamental similarities. In particular, people who look alike were perceived as being similar more generally. Interestingly, they were also thought to share similar preferences and common knowledge, indicating that children inferred similarities in various mental states from observing similarity in the way people look. These findings thus extend previous work showing that children perceive members of their *own* social categories as similar to themselves and expect them to share the same preferences [46, 47] by demonstrating that they make similar judgments about third-party social categories more generally.

Children judged the *loose association* to stand out with regard to its characteristics of a group as a whole. That is, people who happen to stand at the same tram stop were perceived to have a lack of continuance (i.e., they were unlikely to meet again). In addition, they were expected to have low permeability, meaning children thought this group would be particularly difficult to join. At first glance this is somewhat surprising, as, according to Lickel and colleagues [3], such a transient group should theoretically be one that people can easily join and leave, a judgment commonly made in adults. Interestingly, children frequently justified their assessment by saying that one could not join these people at the tram stop because they were not an actual group (e.g., “…because they don’t belong together” or “…because they are strangers”), echoing their evaluation in the entitativity trials (see above).

These results suggest that children as young as 5 years of age show the origins of an intuitive group typology that is similar to that of adults. The set of group characteristics we chose to ask about was based broadly on previous studies with adults and 10-year-olds [3, 7], with additions that were relevant for the literature on young children. Given these and other differences in the procedures across studies (such as the use of a simplified forced-choice task in the current study instead of complex sorting and rating measures), a direct comparison of the judgments of the young children in this study and those of adults and older children in previous studies is not possible. However, some general parallels besides the evaluation of entitativity discussed above can be drawn. As Bennett [22] notes, adults’ evaluations of groups are based on more underlying and abstract features than are those of children, who tend to focus on characteristics that are easier to observe from outside (see also [48, 49]). For example, adults describe members of *intimacy groups* as being interdependent with and similar to each other. Both the younger children in this study as well as the older children studied by Svirydzenka and colleagues [7] seemed not to share this conception, presumably because the interdependent relationship and similarities of friends, for example, are not as straightforward and easy to observe as the interdependence of a *task group*, or the similarity between members of a *social category* (who often share observable markers such as similar clothing, skin color, or language).

In this study, we presented children with four types of groups, but it is possible that preschoolers might distinguish even more than these four basic types, or might have a more fine-grained perception of subtypes within these basic types. This needs to be examined in further studies. One limitation of this study is that for practical reasons we only asked about one prototype of each type of group. However, we would expect very similar findings on many of the group characteristics questions for other prototypes. For example, Olson and Spelke [20] have shown that children direct others to share equally with both friends and kin (two different examples of *intimacy groups*), and the studies finding enhanced helping of and sensitivity to the interdependence of *task group* members used various examples of task group contexts [21, 43, 50, 51]. It is less clear at the moment whether children would expect different examples of *social category* members (e.g., race, language, gender, minimal groups) to be as similar to each other as in the current study. Previous studies show that children respond differently to different examples of social categories [13, 52], so their expectations about different examples of social category group members might well vary. This needs to be investigated in future research.

In summary, for 5- to 6-year-olds, not all groups are the same. By this age, children are beginning to distinguish the same four key types of groups as adults: They judge them to be different in nature, and associate different patterns of characteristics with each group type. This study thus demonstrates how deeply rooted our folk group typology is. Holding different intuitive theories about different types of groups likely influences not only how children perceive groups, but also how they behave within groups, and how they understand and predict both intra- and inter-group interactions. This study therefore casts new light on children's intuitive understanding of groups and group members' relationships and has implications for theoretical accounts of the origins of group psychology and thus the nature of the mature social mind.

## Supporting Information

S1 Data Full anonymized dataset. (XLSX)

## References

[1] Hirschfeld LA. On a folk theory of society: Children, evolution, and mental representations of social groups. Personality and Social Psychology Review. 2001;5(2):107–17. doi: 10.1207/S15327957pspr0502_2 .

[2] Lickel B, Hamilton DL, Sherman SJ. Elements of a lay theory of groups: Types of groups, relational styles, and the perception of group entitativity. Personality and Social Psychology Review. 2001;5(2):129–40. doi: 10.1207/s15327957pspr0502_4

[3] Lickel B, Hamilton DL, Wieczorkowska G, Lewis A, Sherman SJ, Uhles AN. Varieties of groups and the perception of group entitativity. Journal of personality and social psychology. 2000;78:223–46. doi: 10.1037/0022-3514.78.2.223 10707331 . 10707331 10.1037//0022-3514.78.2.223

[4] Campbell DT. Common fate, similarity, and other indexes of the status of aggregates of persons as social entities. Behav Sci. 1958;3(1):14–25. .

[5] Brewer MB, Harasty AS. Seeing groups as entities: The role of perceiver motivation In: Sorrentino RM, Higgins ET, editors. Handbook of motivation and cognition, Vol 3: The interpersonal context. 3. New York, NY: Guilford Press; 1996 p. 347–70.

[6] Hamilton DL, Sherman SJ, Lickel B. Perceiving social groups: The importance of the entitativity continuum In: Sedikides C, Schopler J, Insko CA, editors. Intergroup cognition and intergroup behavior. Mahwah, NJ, US: Lawrence Erlbaum Associates Publishers; 1998 p. 47–74.

[7] Svirydzenka N, Sani F, Bennett M. Group entitativity and its perceptual antecedents in varieties of groups: A developmental perspective. European Journal of Social Psychology. 2010;40:611–24. doi: 10.1002/ejsp.761 .

[8] Fiske AP. The 4 elementary forms of sociality—Framework for a unified theory of social-relations. Psychol Rev. 1992;99(4):689–723. doi: 10.1037//0033-295x.99.4.689 . 1454904 10.1037/0033-295x.99.4.689

[9] Fiske AP. Structures of social life: The four elementary forms of human relations: Communal sharing, authority ranking, equality matching, market pricing New York: Free Press; 1991.

[10] Lickel B, Rutchick AM, Hamilton DL, Sherman SJ. Intuitive theories of group types and relational principles. Journal of Experimental Social Psychology. 2006;42(1):28–39. doi: 10.1016/j.jesp.2005.01.007 .

[11] Kinzler KD, Dupoux E, Spelke ES. The native language of social cognition. P Natl Acad Sci USA. 2007;104(30):12577–80. doi: 10.1073/pnas.0705345104 . 10.1073/pnas.0705345104 PMC1941511 17640881

[12] Kinzler KD, Spelke ES. Do infants show social preferences for people differing in race? Cognition. 2011;119(1):1–9. Epub 2011/02/22. doi: 10.1016/j.cognition.2010.10.019 ; PubMed Central PMCID: PMC3081609. 21334605 10.1016/j.cognition.2010.10.019 PMC3081609

[13] Dunham Y, Baron AS, Carey S. Consequences of "minimal" group affiliations in children. Child Development. 2011;82(3):793–811. doi: 10.1111/j.1467-8624.2011.01577.x . 21413937 10.1111/j.1467-8624.2011.01577.x PMC3513287

[14] Patterson MM, Bigler RS. Preschool children's attention to environmental messages about groups: Social categorization and the origins of intergroup bias. Child Development. 2006;77(4):847–60. doi: 10.1111/j.1467-8624.2006.00906.x 16942493 . 16942493 10.1111/j.1467-8624.2006.00906.x

[15] Waxman SR. Names will never hurt me? Naming and the development of racial and gender categories in preschool-aged children. European Journal of Social Psychology. 2010;40(4):593–610. doi: 10.1002/ejsp.732 .

[16] Diesendruck G, haLevi H. The role of language, appearance, and culture in children's social category-based induction. Child Development. 2006;77(3):539–53. doi: 10.1111/j.1467-8624.2006.00889.x 16686787. . 16686787 10.1111/j.1467-8624.2006.00889.x

[17] McGlothlin H, Killen M. Intergroup attitudes of European American children attending ethnically homogeneous schools. Child Development. 2006;77(5):1375–86. doi: 10.1111/j.1467-8624.2006.00941.x 16999805 . 16999805 10.1111/j.1467-8624.2006.00941.x

[18] Rhodes M. Naïve theories of social groups. Child Development. 2012;83(6):1900–16. Epub 2012/08/22. doi: 10.1111/j.1467-8624.2012.01835.x . 22906078 10.1111/j.1467-8624.2012.01835.x

[19] McGlothlin H, Killen M, Edmonds C. European-American children's intergroup attitudes about peer relationships. British Journal of Developmental Psychology. 2005;23:227–49. doi: 10.1348/02151005x26101 .

[20] Olson KR, Spelke ES. Foundations of cooperation in young children. Cognition. 2008;108(1):222–31. doi: 10.1016/j.cognition.2007.12.003 . 18226808 10.1016/j.cognition.2007.12.003 PMC2481508

[21] Plötner M, Over H, Carpenter M, Tomasello M. The effects of collaboration and minimal-group membership on children’s prosocial behavior, liking, affiliation, and trust. J Exp Child Psychol. 2015;139:161–73. doi: 10.1016/j.jecp.2015.05.008 26112747 10.1016/j.jecp.2015.05.008

[22] Bennett M. Children's social identities. Infant and Child Development. 2011;20(4):201–22. doi: 10.1002/icd.741 .

[23] Dunham Y, Emory J. Of affect and ambiguity: The emergence of preference for arbitrary ingroups. Journal of Social Issues. 2014;70(1):81–98. doi: 10.1111/josi.12048 .

[24] Aboud FE. Children and prejudice New York, NY: Basil Blackwell; 1988.

[25] Nesdale D. The development of ethnic prejudice in early childhood: Theories and research In: Saracho ON, Spodek B, editors. Contemporary perspectives on socialization and social development in early childhood education. Charlotte, NC: IAP Information Age Publishing; US; 2007 p. 213–40.

[26] Dunham Y, Baron AS, Banaji MR. The development of implicit intergroup cognition. Trends in Cognitive Science. 2008;12(7):248–53. Epub 2008/06/17. doi: 10.1016/j.tics.2008.04.006 . 18555736 10.1016/j.tics.2008.04.006

[27] Rhodes M. How two intuitive theories shape the development of social categorization. Child Dev Perspect. 2013;7(1):12–6. doi: 10.1111/cdep.12007 .

[28] Kalish CW, Lawson CA. Development of social category representations: Early appreciation of roles and deontic relations. Child Development. 2008;79(3):577–93. doi: 10.1111/j.1467-8624.2008.01144.x . 18489414 10.1111/j.1467-8624.2008.01144.x

[29] Diesendruck G, Eldror E. What children infer from social categories. Cognitive Development. 2011;26(2):118–26. doi: 10.1016/j.cogdev.2010.11.001 .

[30] Misch A, Over H, Carpenter M. Stick with your group: Young children’s attitudes about group loyalty. J Exp Child Psychol. 2014;126:19–36. doi: 10.1016/j.jecp.2014.02.008 24842584 10.1016/j.jecp.2014.02.008

[31] Shutts K, Roben CKP, Spelke ES. Children's use of social categories in thinking about people and social relationships. Journal of Cognition and Development. 2013;14(1):35–62. doi: 10.1080/15248372.2011.638686 . 23646000 10.1080/15248372.2011.638686 PMC3640585

[32] Gelman SA. Psychological essentialism in children. Trends in cognitive sciences. 2004;8(9):404–9. doi: 10.1016/j.tics.2004.07.001 . 15350241 10.1016/j.tics.2004.07.001

[33] Diesendruck G, Markson L. Children's assumption of the conventionality of culture. Child Dev Perspect. 2011;5(3):189–95. doi: 10.1111/j.1750-8606.2010.00156.x .

[34] Giraudoux P. pgirmess: Data analysis in ecology. [R package version 1.5.7.] 2013.

[35] Zar JH. Biostatistical analysis 4th ed. New Jersey: Prentice Hall; 1999.

[36] Manly BFJ. Randomization, bootstrap and Monte Carlo methods in biology New York: Chapman & Hall; 1997.

[37] Adams DC, Anthony CD. Using randomization techniques to analyse behavioural data. Animal Behaviour. 1996;51:733–8. .

[38] Bigler RS, Liben LS. A developmental intergroup theory of social stereotypes and prejudice In: Kail RV, editor. Advances in child development and behavior. 34. San Diego, CA: Elsevier Academic Press; US; 2006 p. 39–89. 10.1016/s0065-2407(06)80004-2 17120802

[39] Tomasello M, Melis AP, Tennie C, Wyman E, Herrmann E. Two key steps in the evolution of human cooperation. Curr Anthropol. 2012;53(6):673–92. doi: 10.1086/668207 .

[40] Trivers RL. Evolution of reciprocal altruism. Q Rev Biol. 1971;46(1):35–57. doi: 10.1086/406755 .

[41] Bratman ME. Shared cooperative activity. The Philosophical Review. 1992;101:327–41.

[42] Moore C. Fairness in children's resource allocation depends on the recipient. Psychological Science. 2009;20(8):944–8. doi: 10.1111/j.1467-9280.2009.02378.x 19515118 . 19515118 10.1111/j.1467-9280.2009.02378.x

[43] Hamann K, Warneken F, Tomasello M. Children's developing commitments to joint goals. Child Development. 2012;83(1):137–45. Epub 2011/12/17. doi: 10.1111/j.1467-8624.2011.01695.x . 22172281 10.1111/j.1467-8624.2011.01695.x

[44] Hamann K, Warneken F, Greenberg JR, Tomasello M. Collaboration encourages equal sharing in children but not in chimpanzees. Nature. 2011;476(7360):328–31. Epub 2011/07/22. doi: 10.1038/nature10278 . 21775985 10.1038/nature10278

[45] Warneken F, Chen F, Tomasello M. Cooperative activities in young children and chimpanzees. Child Development. 2006;77(3):640–63. doi: 10.1111/j.1467-8624.2006.00895.x 16686793 . 16686793 10.1111/j.1467-8624.2006.00895.x

[46] Bennett M, Sani F. Children's subjective identification with social groups: A self-stereotyping approach. Developmental Science. 2008;11(1):69–75. doi: 10.1111/j.1467-7687.2007.00642.x 18171369 . 18171369 10.1111/j.1467-7687.2007.00642.x

[47] McGraw KO, Durm MW, Durnam MR. The relative salience of sex, race, age, and glasses in children's social perception. Sep 1989. The Journal of Genetic Psychology: Research and Theory on Human Development. 1989;150(3):251–67. doi: 10.1080/00221325.1989.9914595 . 10.1080/00221325.1989.9914595 2809573

[48] Quintana SM. Children's developmental understanding of ethnicity and race. Applied & Preventive Psychology. 1998;7(1):27–45. doi: 10.1016/S0962-1849%2898%2980020–6 .

[49] Livesley WJ, Bromley DB. Person perception in childhood and adolescence Oxford, England: John Wiley & Sons; 1973.

[50] Gräfenhain M, Behne T, Carpenter M, Tomasello M. Young children's understanding of joint commitments. Developmental Psychology. 2009;45(5):1430–43. doi: 10.1037/A0016122 . 19702403 10.1037/a0016122

[51] Gräfenhain M, Carpenter M, Tomasello M. Three-year-olds' understanding of the consequences of joint commitments. Plos One. 2013;8(9):e73039 doi: k10.1371/journal.pone.0073039 . 24023805 10.1371/journal.pone.0073039 PMC3762880

[52] Kinzler KD, Shutts K, Dejesus J, Spelke ES. Accent trumps race in guiding children's social preferences. Social Cognition. 2009;27(4):623–34. doi: 10.1521/soco.2009.27.4.623 21603154 . 21603154 10.1521/soco.2009.27.4.623 PMC3096936

